# Altered RECQL5 expression in urothelial bladder carcinoma increases cellular proliferation and makes RECQL5 helicase activity a novel target for chemotherapy

**DOI:** 10.18632/oncotarget.12683

**Published:** 2016-10-15

**Authors:** Karl Patterson, Lovleen Arya, Sarah Bottomley, Susan Morgan, Angela Cox, James Catto, Helen E. Bryant

**Affiliations:** ^1^ Academic Unit of Molecular Oncology, Sheffield Institute for Nucleic Acids (SInFoNiA), Department of Oncology and Metabolism, University of Sheffield, Sheffield S10 2RX, United Kingdom; ^2^ Academic Unit of Urology, Sheffield Institute for Nucleic Acids (SInFoNiA), Department of Oncology and Metabolism, University of Sheffield, Sheffield S10 2RX, United Kingdom

**Keywords:** RECQL5, helicase, bladder cancer, targeted therapy

## Abstract

RECQ helicases are a family of enzymes with both over lapping and unique functions. Functional autosomal recessive loss of three members of the family BLM, WRN and RECQL4, results in hereditary human syndromes characterized by cancer predisposition and premature aging, but despite the finding that RECQL5 deficient mice are cancer prone, no such link has been made to human RECQL5. Here we demonstrate that human urothelial carcinoma of the bladder (UCC) has increased expression of RECQL5 compared to normal bladder tissue and that increasing RECQL5 expression can drive proliferation of normal bladder cells and is associated with poor prognosis. Further, by expressing a helicase dead RECQL5 and by depleting bladder cancer cells of RECQL5 we show that inhibition of RECQL5 activity has potential as a new target for treatment of UCC.

## INTRODUCTION

Urothelial carcinoma of the bladder (UCC) is the fourth most common cancer in men and seventh most common in women, affecting more than 400,000 people world wide annually [1]. Around 1/3 of cancers are high grade and have a poor prognosis when invading into the bladder wall. The median survival for muscle invasive cancers is around 50% and has not improved in recent years, since the introduction of platinum based therapies [2–4]. Currently there is no treatment able to increase survival after platinum failure, thus there is an urgent need to develop new and effective agents. Molecular changes such as altered gene expression, have potential as targets to increase therapeutic response to conventional chemotherapies or as stand-alone agents. There has been some success in identifying molecular targets for therapy in UCC [5–7], which have lead to several current clinical trials [8, 9], however to date none have progressed to standard care.

Genomic stability plays a key role in preventing tumourigenesis and instability is a key hallmark of cancer [10]. Alterations in the pathways responsible for maintain genomic instability can influence tumour development and response to therapy, while the same pathways can be themselves targets for therapy [11–15]. The RECQ helicases are conserved throughout evolution and contribute to genome stability through roles in replication, base excision repair, double-strand break repair, transcription, telomere maintenance and mitochondrial function [16]. All family members share a homologous helicase and RecQ C-Terminal domain, which contains a zinc-binding motif, a helix-hairpin-helix winged-helix domain and a β-hairpin motif. There are 5 mammalian RECQ helicases, RECQL (RECQ1), BLM (RECQ2), WRN (RECQ3), RECQL4, and RECQL5, having some over lapping and some distinct functions. Functional autosomal recessive loss of BLM, WRN and RECQL4, results in hereditary human syndromes characterized by cancer predisposition and premature aging (Bloom's, Werner's, and Rothmund-Thomson's syndromes, respectively) [17]. Further a number of polymorphisms are identified for the RECQ helicases that can determine survivability and susceptibility to cancers [18–24] and recently we demonstrated that increased expression of RECQL5 can be associated to poor prognosis in breast cancer [25]. While no hereditary disease is associated with RECQL5, deficient mice display high levels of spontaneous double strand breaks, are susceptible to gross chromosomal rearrangements and are prone to develop lymphomas and various solid tumours [26, 27], suggesting RECQL5 could also play a role in preventing tumourigenesis. In support of this RECQL5 is reported to have roles in suppression or repair of endogenous DNA damage [28–30], and in overcoming/repairing damage induced by DNA crosslinks [31–34], thymidine [35], hydroxyurea [33], camptothecin [34, 36] and transcription-replication collisions [37–40]. RECQL5 has 3 isoforms α, β and γ. While all 3 isoforms contain the helicase domain only the β isoform is nuclear [41] and it is this form which is generally considered as being involved in the cellular DNA damage response pathway. For the most part in this work specifically RECQL5β is considered while reference to RECQL5 infers no specific isoform is being implicated.

We demonstrate that UCC have increased expression of RECQL5β compared to normal bladder tissue and that increasing RECQL5β expression can drive proliferation of normal bladder cells. Further by using a helicase dead RECQL5β we show that inhibition of RECQL5 activity is a potential new target for treatment of UCC.

## RESULTS

### Primary UCCs have significantly increased expression of RECQL5 helicase protein

**Figure 1:**
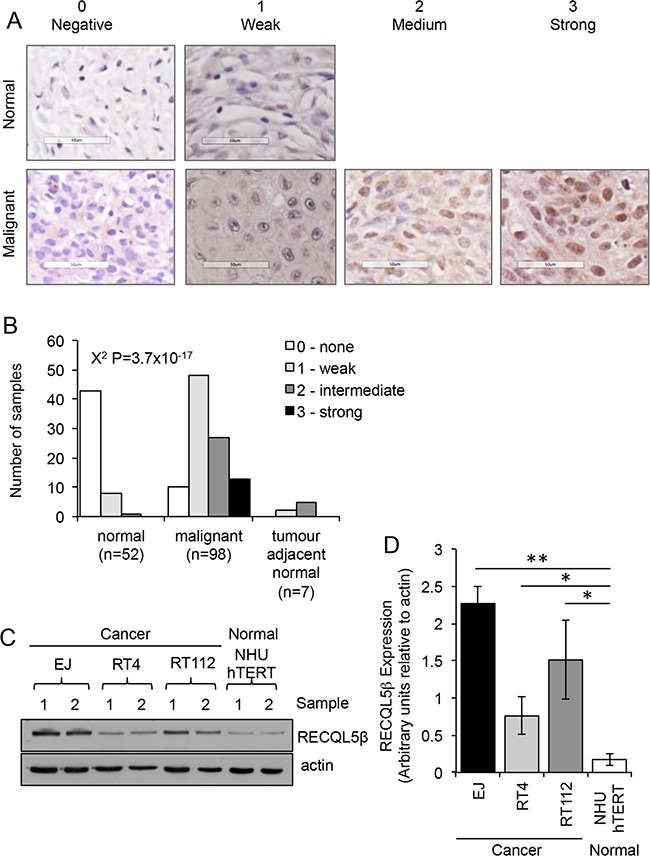
RECQL5 protein expression is increased in UCC **A.** Tissue microarray stained for RECQL5β and scored on a scale of 0-3 depending on whether the nuclear staining was negative, weak, medium, or strong respectively. Examples of normal and malignant tissue in each category are shown. None of the normal samples had medium or strong staining. **B.** Quantification of staining and statistical significance calculated using Contingency Table Pearson Chi-square test. **C.** Western blot for RECQL5β in normal immortalised bladder (NHU hTERT) and UCC (EJ, RT4, and RT112) cells; 2 successive passages of the cells are shown. **D.** Quantification of western blots average and standard deviation of 4 biological repeats is shown, * and ** indicate p<0.05 and p<0.01 respectively using Students t-test.

**Figure 2:**
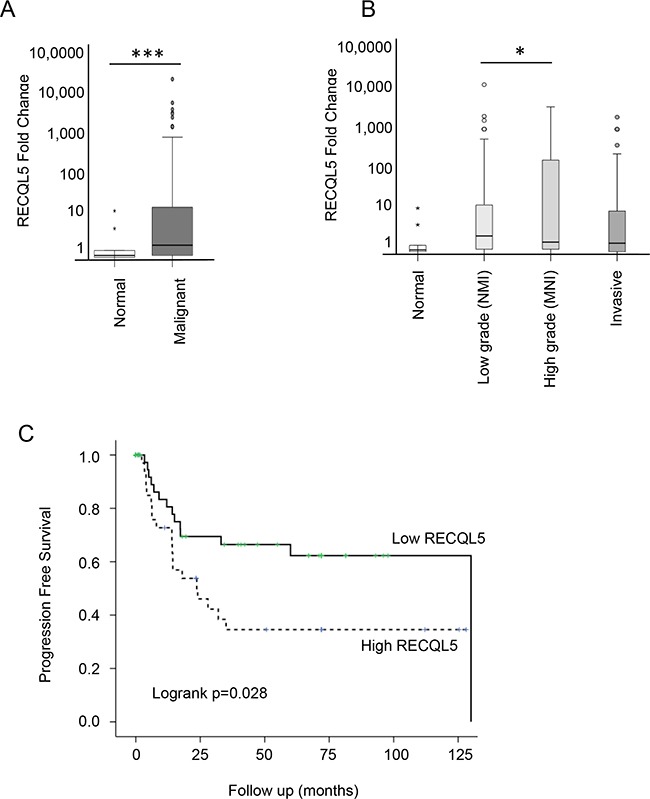
RECQL5 mRNA is increased in UCC and is associated with poor prognosis Taqman qRT-PCR quantification of mRNA from 197 primary bladder tumour and 20 normal tissue samples using a RECQL5 probe and plotted as fold change [52]. **A.** Stratified by normal compared to malignant tissue and **B.** according to tumour grade; * and *** indicate p<0.05 and p<0.001 respectively using Students t-test **C.** Kaplan Meier curve showing progression free survival based on *RECQL5* mRNA expression, p value calculated as log-rank score.

Expression of RECQL5 helicase protein was analysed in 98 sporadic primary human UCC samples and 52 normal bladder tissue (Supplementary Table S1) using an antibody specific to RECQL5β (Supplementary Figure S1). Tissues were scored for strength of nuclear staining (Figure 1A). There was a highly significant increase in nuclear staining of RECQL5 in the malignant samples (Figure 1B, Contingency Table Pearson Chi-square test P=3.7×10e-17), with 90% of the malignant bladder samples scoring positive for RECQL5β compared to normal bladder samples where 83% of samples were negative for any nuclear staining. Tumour Samples were then grouped by stage and grade and RECQL5 intensity compared (Supplementary Figure S2). No significant difference in degree of RECQL5β expression was seen between tumour samples of differing stage (Contingency Table Pearson Chi-square test P=0.953), and suggestive evidence of expression with higher-grade metastatic disease was seen but only when samples were grouped by staining vs. non-staining (Contingency Table Pearson Chi-square test P = 0.035). A small number of tumour adjacent normal samples were also analysed (Figure 2), no significant difference was detected most likely due to sample size however a trend to more RECQL5 staining compared to normal can be argued. Interestingly a few malignant samples (n=3) and no normal samples displayed additional cytoplasmic staining for RECQL5β, however numbers are too low to determine significance (data not shown).

Consistent with this western blotting revealed that three UCC cell lines, EJ, RT112 and RT4, have significantly higher levels of RECQL5β protein expression when compared to an immortalised normal bladder cell line, NHU hTERT (Figure 1C&1D - Student's t-test, p values = 2.1 × 10^−3^, 4.4 × 10^−2^ and 4.1 × 10^−2^ respectively).

The number of RECQL5 foci in cells has been shown to vary throughout the cell cycle and it's expression is moderately increased following replication stress [35]. However when comparing UCC and normal bladder cell lines there was no significant difference in the percentage of cells in each phase of the cell cycle (Supplementary Figure S3) suggesting that differences in expression are tumour specific rather than cell cycle related. To further examine this in patient samples, a number of UCC tumour sections were stained for Ki67 in parallel to RECQL5; high RECQL5 staining could be seen with or without Ki67 staining, confirming that RECQL5 expression in tumours is not due only to increased cellular proliferation (Supplementary Figure S4). Finally in support of the change being unrelated altered proliferation, previous studies where RECQL5 was knocked out using gene distruption in DT40 cells, pulse labeling with BrdU staining demonstrated that the fraction of cells in S-phase is unaltered [32].

### Aggressive UCC has significantly increased levels of *RECQL5* mRNA, which are associated with worse survival

Expression of *RECQL5* mRNA was analysed in 197 primary UCC samples and 20 normal tissue samples (Supplementary Table S2). There was a significant increase in RECQL5 mRNA levels in the bladder cancers compared to the normal bladder tissue (Figure 2A – Student's t.test p<0.001). A similar but less significant (p=0.03) result was found when using a probe that specifically recognises *RECQL5β* (data not shown). When tumours were stratified by grade, *RECQL5* expression seemed highest in those of high grade but that were not yet invasive (Figure 2B – Student's t.test p=0.04). This is consistent with survival analysis whereby high *RECQL5* mRNA was associated with reduced progression free survival (Figure 2C – log-rank test p=0.028).

### Depletion of RECQL5 specifically reduces proliferation of UCC cells

**Figure 3:**
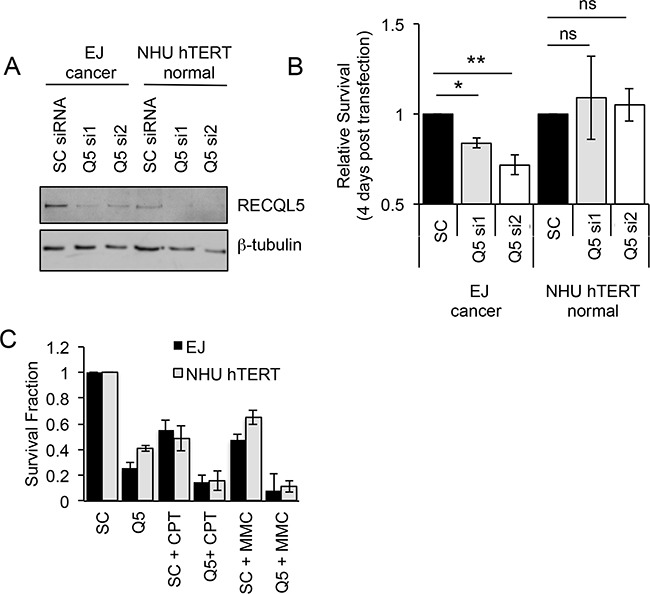
Depletion of RECQL5 reduces survival in bladder cancer cells **A.** Western blot for RECQL5β of UCC (EJ) and immortalised normal bladder (NHU hTERT) cells depleted of RECQL5 using siRNA against RECQL5β specifically (Q5 si1) or all 3 isoforms of RECQL5 (Q5 si2) compared to cells treated with non-targeting control (SC) siRNA. **B.** Survival fraction of corresponding RECQL5 depleted cells 96 h post siRNA transfection. **C.** Survival fraction of siRNA treated cells treated with or without 25 nM camptothecin (CPT) or 100 nM mitomycin C (MMC). Average and standard deviation of at least 3 experiments is shown, * and ** indicate p<0.05 and p<0.01 respectively using Students t-test, ns = not significant.

Cancer specific overexpression of a protein represents a potential site of therapeutic intervention. To test whether RECQL5 is such a target, UCC and normal bladder cells were depleted of RECQL5 protein using siRNA (Figure 3A). Ninety six hours post transfection the relative survival of bladder cancer cells depleted of either all isoforms of RECQL5 (si1) or specifically RECQL5β (si2) was significantly reduced (Student's t-test, p value = 5.1 × 10^−3^ and 3.5 × 10^−3^ respectively), while the survival of normal bladder cells was not affected (Figure 3B), suggesting that RECQL5 could be a cancer specific target for therapeutic intervention in UCC. It has been reported that RECQL5 is involved in recovery from DNA cross-links [31–34] and camptothecin [34, 36] induced DNA damage. Here depletion of RECQL5 (using a pool of RECQL5 si1 and si2) reduced UCC cell survival following treatment with the DNA cross-linking agent MMC or the topoisomeraise inhibitor camptothecicn compared to treatment with the DNA damaging alone (Figure 3C), suggesting combination therapy could be beneficial.

### Exogenous expression of a helicase dead RECQL5 reduces proliferation and sensitizes to replication stress in UCC cells while expression of RECQL5 increases proliferation in normal bladder cells

**Figure 4:**
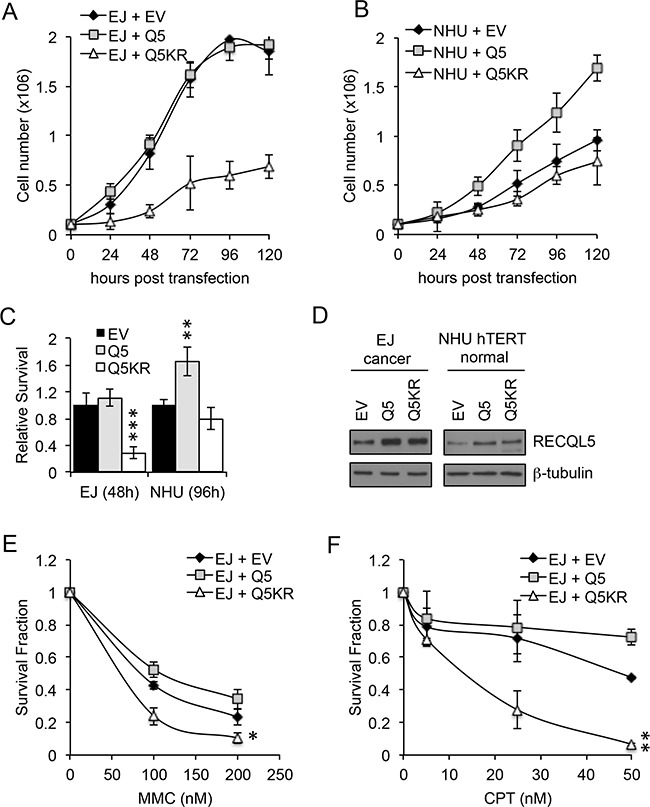
Exogenous expression of RECQL5β increases proliferation in normal bladder cells and expression of helicase dead RECQL5 **A** and **C.** Normal immortalised bladder (NHU hTERT) cells and UCC (EJ) cells transiently transfected with empty vector (MOCK), wildtype RECQL5β (Q5) or helicase dead RECQL5β (Q5KR) and western blotted for RECQL5β. **B** and **D.** Cell number in each of the transfected conditions after 24, 48, 72, 96 and 120 h. Average and standard deviation of 3 independent repeats is shown. Significance was calculated using a student's t-test, ***indicates p<0.001 comparing MOCK to Q5KR transfected EJ cells at 120 h post-transfection and **indicates p<0.01 comparing MOCK to Q5 transfected NHU hTERT cells at 120 h. **E** and **F.** Clonogenic survival fraction of EJ cells treated as above for 48 h then replated into mitomycin C (MMC) or camptothecin (CPT). Average and standard deviation of 3 independent repeats is shown. Significance was calculated using a student's t-test, **indicates p<0.01 and *indicates p<0.05 comparing Q5 to Q5KR transfected EJ cells.

UCC and normal bladder cells were transiently transfected with wildtype (Q5) or helicase dead (Q5KR) RECQL5β (Figure 4D). As UCC cells proliferate faster than normal bladder cells the relative effect of expression of Q5 and Q5KR was compared after 3 cell doublings which corresponded to 48 h for UCC and 96 h for normal bladder cells. Proliferation of Q5KR transfected UCC cells (EJ) was significantly decreased compared to Q5 or empty vector (EV) transfected cancer cells (Figure 4A&4C) (Student's t-test, p value = 5.1 × 10^−4^ – Q5KR c.f. EV at 48 h). This effect was not seen in normal cells (NHU) (Figure 4B&4C). In contrast to the helicase dead Q5 ectopic expression of wildtype Q5 in UCC cells did not alter proliferation compared to EV transfected cells (Figure 4A&4C), while it's expression in non-tumour NHU hTERT cells significantly increased proliferation compared to EV or Q5KR transfected cells (Figure 4B&4C) (Student's t-test, p value = 4 × 10^−3^ – Q5 c.f. EV at 96 h).

UCC cells were also transfected with Q5 or Q5KR, then replated into MMC or camptothecin for 10 days. Colony survival assays, normalised for effect of ectopic expression Q5/Q5KR alone, showed that in this cell line over expression of Q5KR can sensitize to DNA damaging agents (Figure 4E and 4F) (Student's t-test, p value = 0.014 MMC and p=0.0015 camptothecicn – Q5KR c.f. Q5).

These data again suggest that RECQL5β helicase activity maybe a valid target for specific killing of UCC and for sensitising UCC to conventional chemotheraputics. Further they provide evidence that overexpression of RECQL5 may contribute to increased proliferation and thus cellular transformation in bladder cells.

## DISCUSSION

To date no inherited cancer predisposition syndrome has been associated with RECQL5. However reports of alteration in RECQL5 in sporadic cancer are emerging, these include polymorphisms associated with cancer [19, 22–24], one report that *RECQL5* mRNA and protein levels are reduced in colorectal cancer (CRC) [42] and a recent report by our lab showing high levels of RECQL5 are associated with aggressive phenotypes and poor survival outcomes in breast cancer [25]. In contrast to CRC, here *RECQL5* mRNA and nuclear RECQL5β protein were increased in UCC when compared to normal tissue. This is slightly unexpected given that RECQL5 deficient mice are cancer-prone and suggests that, like several other members of the RECQ helicase family [25, 43], increased expression of RECQL5 can also be associated with cancer. Normal bladder cells could be induced to proliferate by exogenous expression of wildtype but not helicase dead RECQL5β demonstrating a potential functional role of increased RECQL5β expression in cellular transformation. Previously overexpression of RECQL5β has been shown to allow cells to overcome replication stress [35] and it is possible that an ability to overcome replication stress gives cells a selective advantage during tumourigenesis. However the consequence of doing so maybe genomic instability, as overexpression of RECQL5β also reduces replication stress induced checkpoint activation and homologous recombination [35]. Interesting in breast cancer we previously showed that high RECQL5 protein level needed to be associated with low RAD51 protein expression to infer poor survival, suggesting a functional relationship between RECQL5 and RAD51 pathways influences breast cancer tumour progression or response to therapy [25].

Consistent with findings in breast cancer [25] increased *RECQL5* mRNA was associated with poor prognosis in bladder cancer, suggesting value as a prognostic marker, perhaps with particular value in identifying high-grade tumours which are likely to progress to more serious disease. In addition RECQL5β protein expression tended to have stronger staining in higher-grade metastatic disease.

Exogenous expression of RECQL5β in normal bladder cells increased proliferation, while expression of a helicase dead mutant did not significantly alter growth. In contrast no change in growth was seen when wildtype The The potential of RECQL5 as a therapeutic target has been discussed previously in other cancers [25, 34, 35, 44]. RECQL5β levels were increased in bladder cancer cells, while overexpression of a helicase dead RECQL5 protein or siRNA mediated depletion of RECQL5 resulted in reduced cell survival in UCC cells. In addition depletion of RECQL5 or expression of helicase dead RECQL5 increased cell death in MMC and camptothecin treated UCC cells. Taken together these data demonstrate the potential of RECQL5 as a therapeutic target in UCC. Given that several other RECQ helicases have recently been successfully and specifically targeted by small molecule inhibitors [22, 45], it maybe be possible and timely to develop RECQL5 inhibitors for use as chemotherapeutics either as monotherapies or combined with conventional DNA damaging agents.

In summary we have shown that increased expression of RECQL5 protein occurs and is likely to contribute to tumourigenesis in UCC and that the pharmacological targeting of the helicase activity of RECQL5 is a strong target for future small molecule inhibitor development.

## MATERIALS AND METHODS

### Human UCC tissue microarrays

UCC tissue microarrays (BL1002, BL802a, and BNC12011) were purchased from US Biomax, Inc. TMAs contained 1mm diameter and 5 μM thickness cores from formalin-fixed paraffin-embedded (FFPE) samples, some samples were sparse and could not be analysed. Samples that were analysed are shown in Supplementary Table S1, these included 90 urothelial carcinoma, 4 squamous cell carcinoma, 4 adenocarcinoma, 7 adjacent normal bladder tissues and 52 normal tissues. Further data for cases can be found at http://www.biomax.us/tissue-arrays/Bladder/BL1002, http://www.biomax.us/tissue-arrays/Bladder/BL802a, http://www.biomax.us/tissue-arrays/Bladder/BNC12011 and Supplementary Table S1).

### Patients and tumours

We selected new freshly frozen primary tumours treated at the Royal Hallamshire Hospital Sheffield, UK. TUCC were classified using the 2004 WHO/ISUP criteria and treated according to standard care [46, 47]. For comparison we obtained disease free urothelium from patients undergoing radical prostatectomy for prostate cancer. All tissues were confirmed using H&E sections and micro dissected to obtain cell populations with >80% purity. Following resection, all patients were followed for progression (defined as a second bladder tumour of increased stage or until metastases developed). Patient demographics are summarized in Supplementary Table S1 of supporting information. Collection of samples was performed after obtaining informed consent and within an ethics committee approved collection (South Yorks Ethics committee 10/H1310/73).

### Cell lines

UCC cell lines EJ [48], RT112 [48] and RT4 [49] were grown in DMEM with 10% fetal calf serum. Immortalised normal human urothelium cells [50] were grown in Keratinocyte Serum Free Media (KSFM) supplemented with epidermal growth factor (EGF) (5 ng/ml) and bovine pituitary extract (BPE) (50 ng/ml) (Gibco) with 30 ng/ml of cholera toxin (Sigma-Aldrich Company Ltd, Poole, UK).

### RECQL5 helicase expression using IHC

TMAs were deparaffinised and rehydrated in distilled water. IHC, antigen retrieval was performed using heat-induced epitope retrieval buffer (Sodium Citrate Buffer, 10 mM Sodium Citrate, 0.05 % Tween 20, pH 6). Endogenous peroxidase activity was blocked using 3% H_2_O_2_. Anti-RECQL5 rabbit polyclonal antibody (Sigma-Aldrich; HPA029971) was diluted 1:150 in 2% goat serum, 2% casein in PBS. TMAs were incubated with primary antibody overnight at 4°C. 3,3-Diaminobenzidine substrate was used to visualize RECQ-positive cells. TMAs were counterstained with hematoxylin. Nuclear staining was scored using an intensity score ranging from 0 to 3 (0=negative, 1=weak intensity nuclear staining or staining<30% nuclei, 2 =medium intensity staining or staining <60% nuclei, 3=strong intensity nuclear staining in >60% nuclei.

For optimization of IHC EJ cells depleted of RECQL5 using pooled siRNA as below were fixed in 4% paraformaldehyde/PBS overnight, before PFA was replaced first with 70% ethanol then with 2% agarose- 4%formaldehyde and being embedded in wax and sectioned (Supplementary Figure S1A), an IgG control was also included for these pellets. These demonstrate that when RECQL5 is depleted the signal is reduced indicating specificity of primary antibody to the antigen RECQL5. Second an IgG control was used on a test TMA in parallel to each experiment (Supplementary Figure S1B), demonstrating that on each occasion the staining is dependent on the primary antibody rather than a reaction of the secondary antibody. There is also there is also an internal negative control of fibroblast nuclei within the bladder stroma in the arrays, if staining.

Slides were imaged using an Aperio Slide Scanner. Scoring was performed by a trained pathologist. Significance was calculated using Stata Data Analysis and Statistical Software, Version 12.

### Quantitative real-time PCR

RNA from the UCC and normal urothelial cell lines (NHU, RT4, RT112 and EJ) and primary UCC and normal samples was extracted using the RNeasy Mini Kit (Qiagen, Manchester, UK). Approximately 1 × 10^6^ cells were used and manufacturer's instructions were followed to isolate 5 – 35 μg of total RNA. cDNA was generated from RNA using the Applied Biosciences High Capacity cDNA Reverse Transcription Kit. RT-PCR was performed using TaqMan probe and primers (Applied Biosystems, Grand Island, NY) for RECQL5β (Hs00696986_g1) and RECQL5 non isotype specific (Hs00188633_m1), Heat shock protein 90kDa alpha (cytosolic), class B member 1 (HSP90AB1) (Hs03043878_g1), Testis enhanced gene transcript protein (TEGT) (Hs01012085_m1) and Mitochondrial ATP synthase H+ transporting F1 complex beta subunit (ATP5B) (Hs00969573_mH) with TaqMan Universal PCR Mix (Applied Biosystems) as recommended by the manufacturer. The dissociation curve was then calculated. ΔCT values were calculated normalisation to the median of 4 housekeeping genes TEGT, ATP58, HSP90AB and PCNA chosen from previous reports in bladder cancer [51]. Fold change was calculated using DDCt methods [52]. Outcomes with respect to time were plotted using the Kaplan-Meier method and compared using a Log rank test within SPSS (Vsn. 19.0 SPSS Inc.).

### Western blotting

Cells were lysed in lysis buffer (50 mM HEPES, 150 mM NaCl, 1 mM EDTA, 1 mM EGTA, 10% Glycerol, 1% Triton X100, Complete Protease inhibitor complex (Roche, Welwyn Garden City, UK), Phosphatase inhibitor complex 1 and Phosphatase inhibitor complex 2 (Sigma-Aldrich). 50 μg total protein was resolved on an SDS-PAGE gel and transferred to Hybond ECL membrane (GE Healthcare Life Sciences, Little Chalfont, UK). This membrane was immunoblotted with rabbit anti-RECQL5 (AB91422, 1:2000, Abcam, Cambridge, UK), rabbit anti-eLF2α (9722, Cell signalling technology, Danvers, MA), rabbit anti-eLF2α-pS51 (9721, Cell signalling technology) and rabbit anti-β-ACTIN (A2228, 1:2000, Sigma-Aldrich) or mouse anti-β-tubulin (T8328, 1:1000, Sigma-Aldrich), antibodies in 5% milk overnight. After addition of the appropriate HRP conjugated secondary antibody and further washes, immunoreactive protein was visualised using ECL reagents (GE Healthcare Life Sciences) following manufacturers' instructions. Intensity was analysed using imageJ image processing software.

### Plasmids and siRNA

pcDNA 3.1 plasmids containing human RECQL5β cDNA (Q5) and RECQL5KR (RecQL5β cDNA sequence with the helicase domain disrupted by a K58R point mutation – Q5KR)) were a generous gift from Dr. Pavel Janscak (Institute of Molecular Cancer Research, University of Zurich)

Q5 si1 was a pool of two predesigned siGenome siRNA (RECQL5-01 and RECQL5-03, Thermo Scientific, Winsford, UK) designed to target all 3 isoforms of RECQL5, Q5 si2 was a pool of custom oligos (UGAAGAAGGUGGCCGAUAU) and (CUGCAAAUGUUGUGGUCAA) designed only to target RECQL5β.

### Proliferation assays following overexpression of RECQL5

1×10^5^ cells were plated 24 hours prior to transfection with 2.5 μg of plasmid DNA expressing wildtype RECQL5β (Q5) or helicase dead RECQL5β (Q5KR) [35] using Lipofectamine 2000 (Invitrogen, Grand Island, NY). At various times post transfection cells were trypsinised from replica plates, stained with trypan blue and live cells counted. On each occasion 8 counts of each condition were taken and an average calculated. The experiment was repeated on 3 independent occasions and average and standard deviations calculated.

### Survival assays following siRNA mediated depletion of RECQL5

20 μM siRNA against RECQL5/RECQL5β was reverse transfected into 1×10^5^ cells using lipofectamine RNAiMAX. Cells were left 96 hours before counting as above. Relative survival compared to cells transfected with a scrambled control was calculated. The experiment was repeated on 3 independent occasions and average and standard deviations calculated.

### Survival in the presence of MMC and camptothecin

For analysis of sensitivity to DNA damaging agents, cells were treated as above for 48 h prior to replating into 90 mm dishes in presence of increasing doses of MMC and camptothecin and then left 10 days for colonies to form before staining with 4% methylene blue in 70% methanol and counting. The experiment was repeated on 2 independent occasions and average and standard deviations calculated.

## SUPPLEMENTARY FIGURES AND TABLES




