# Supplementary material for: Altered RECQL5 expression in urothelial bladder carcinoma increases cellular proliferation and makes RECQL5 helicase activity a novel target for chemotherapy
Source: Oncotarget. 2016 Oct 15;7(46):76140–50. doi: 10.18632/oncotarget.12683 (PMC5342802; doi:10.18632/oncotarget.12683)
Supplement: Supplementary file 1 [file oncotarget-07-76140-s001.pdf]

Altered RECQL5 expression in urothelial bladder carcinoma increases cellular proliferation and makes RECQL5 helicase activity a novel target for chemotherapy

SUPPLEMENTARY FIGURES AND TABLES

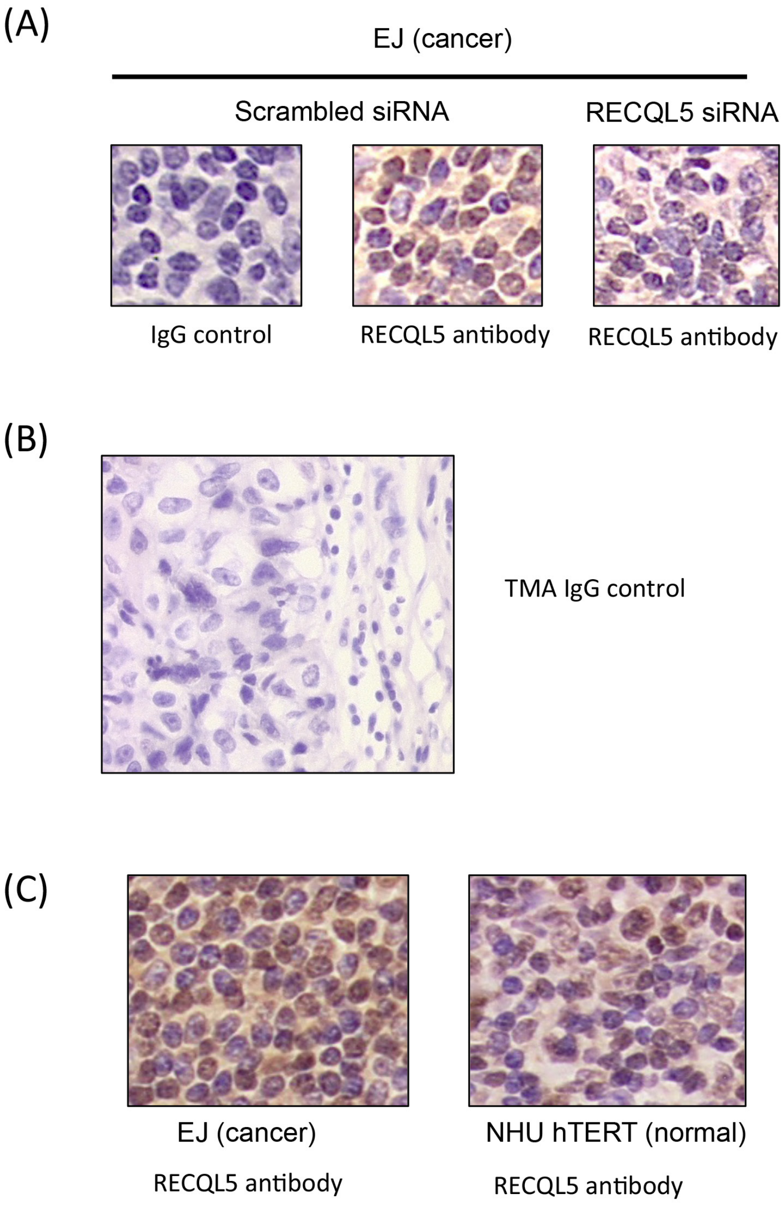

Supplementary Figure S1: RECQL5β expression in bladder cancer and normal bladder cell lines. A. Bladder cancer (EJ) cells) were treated with scrambled or siRNA against RECQL5 for 48h, cell pellets were then harvested, fixed and embedded in wax prior to sectioning and staining with IgG or anti-RECQL5 antibody. Reduced staining after siRNA mediated depletion of RECQL5 and absence of staining with IgG demonstrates the specificity of the antibody. **B.** IgG staining of a sample of the tissue microarray confirms specificity in tumour tissues. **C.** RECQL5 staining of bladder cancer (EJ) and normal bladder (NHU-hTERT) cells confirms western blotting in Figure 1 and supports the hypothesis that bladder cancer expresses higher levels of RECQL5.

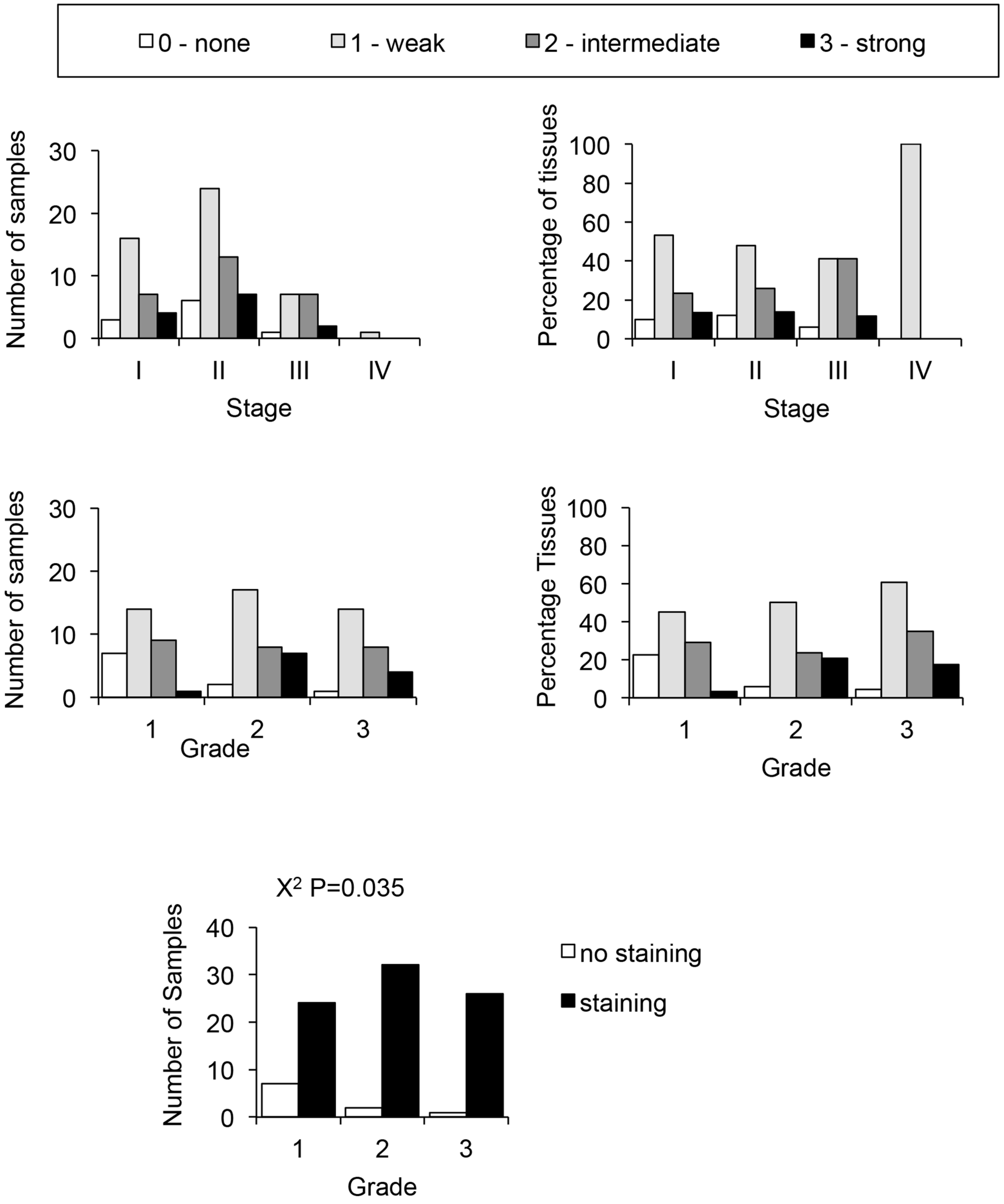

Supplementary Figure S2: RECQL5β expression in primary bladder cancers and normal bladder tissue. A bladder tumour microarray containing 52 normal and 98 tumour histology samples was stained for RECQL5 and scored 0, 1, 2 or 3 depending on whether the nuclear staining was negative, weak, medium, or strong respectively. None of the normal samples had medium or strong staining. **A.** number of metastatic tissues scored at stage I, II, III or IV. **B.** number of metastatic tissues scored at grade 1, 2 or 3. Statistical significance was tested using Contingency Table Chi-squared test.

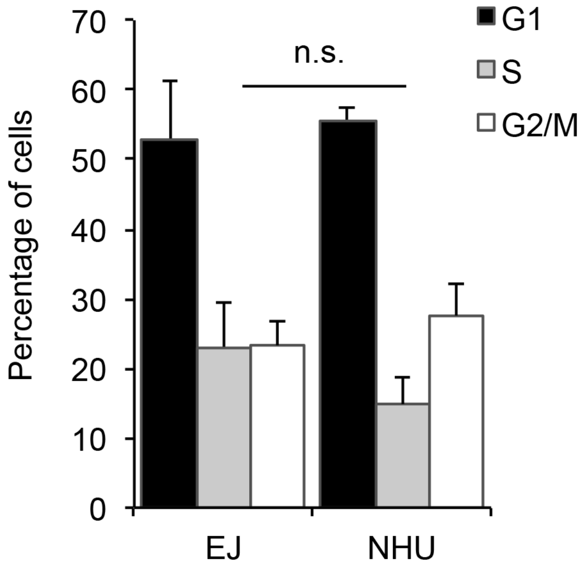

Supplementary Figure S3: Cell cycle analysis of normal and UCC cells. Expotentionaly growing normal bladder (NHU hTERT) and UCC (EJ) cell lines were collected and stained with propidium iodide. No significant difference was seen in cell cycle profile between the cell lines.

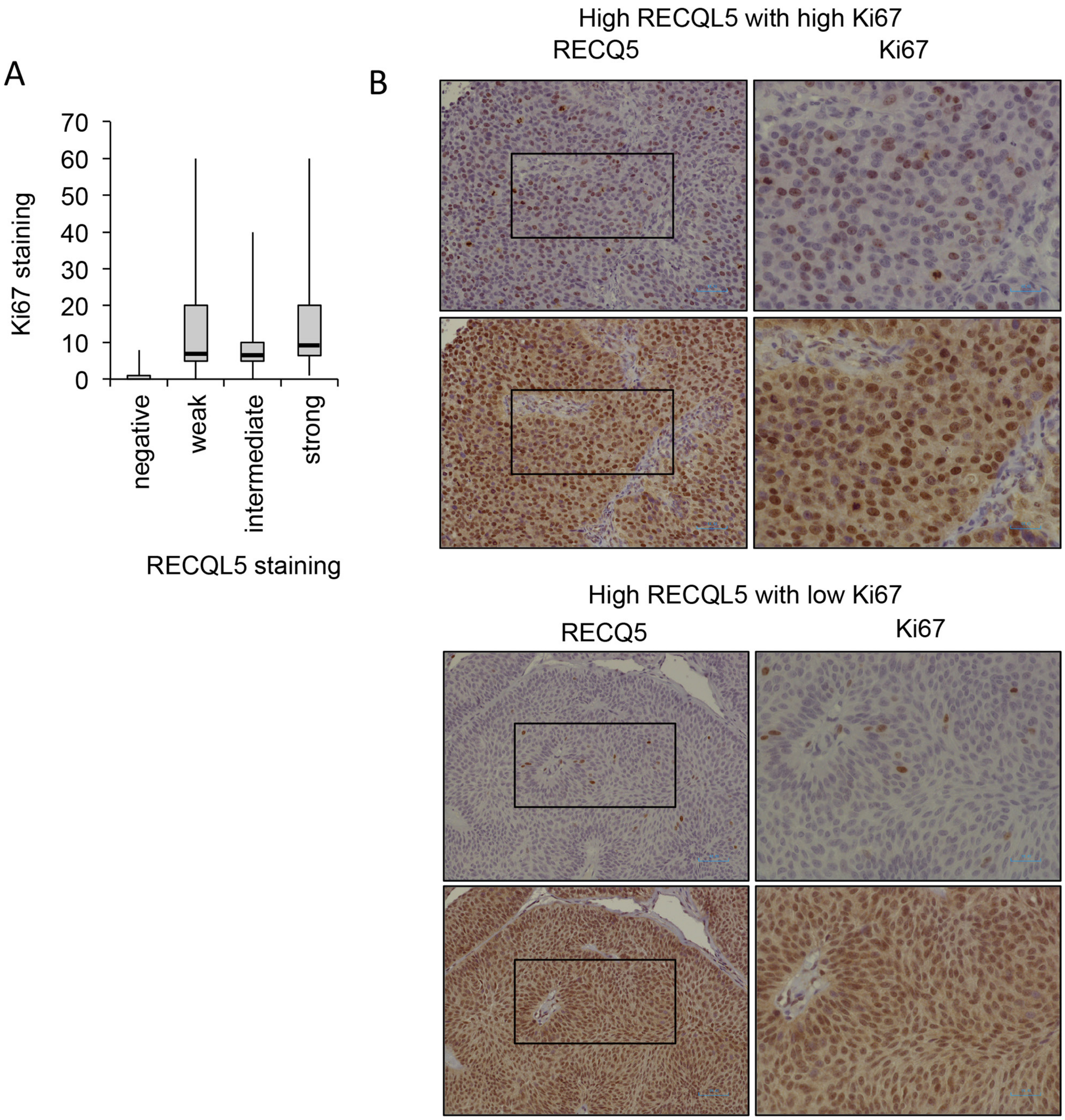

Supplementary Figure S4: Ki67 and RECQL5 staining on human malignant bladder cancer tissue. A subset of bladder cancer sections from Figure 1 (n=60) were also stained for Ki67 and **A.** scored on a scale of 0-100 for percentage positive nuclei across the field, these scores were then plotted on a Box Whisker plot against RECQL5 staining in closely matched sections of the same tissue samples. Tumour samples that were negative for RECQL5 were always associated with very low Ki67. However comparing samples that were weak, medium or strong staining for RECQL5 there was no significant difference in Ki67 staining. **B.** Example of tumour cells strongly stained for RECQL5 displaying different percentages and different staining intensities of Ki67. In each panel the upper images are 40x magnification and the highlighted area is shown at 20x below.

Supplementary Table S1: Patient demographics for IHC analysis

See Supplementary File 1

Supplementary Table S2: Patient demographics for RNA analysis

	number	percentage
**Controls**		
Total	20	
Gender		
Male	20	100%
Female	0	0%
Age (mean and st.dev)	69.4 (+/- 4.3) years	
**Bladder Cancers**		
Total	197	
Gender		
Male	156	79%
Female	41	21%
Age (mean and st.dev)	72.1 (6.7) years	
Histological type		
Urothelial cell carcinoma	197	100%
other	0	
Phenotype		
low grade NMI*	68	35%
high grade NMI	55	27%
invasive	74	39%
Follow up	32 (29) months	
Time (mean and st. dev)		
Stage Progression		
No	36	18%
Yes	115	58%
not known	46	23%

*MNI:Non-muscle invasive.
